# Controlled creation of a singular spinor vortex by circumventing the Dirac belt trick

**DOI:** 10.1038/s41467-019-12787-1

**Published:** 2019-10-16

**Authors:** L. S. Weiss, M. O. Borgh, A. Blinova, T. Ollikainen, M. Möttönen, J. Ruostekoski, D. S. Hall

**Affiliations:** 1 Department of Physics and Astronomy, Amherst College, Amherst, MA 01002–5000 USA; 2 Faculty of Science, University of East Anglia, Norwich, NR4 7TJ UK; 3 Department of Physics, University of Massachusetts, Amherst, MA 01003 USA; 4 QCD Labs, QTF Centre of Excellence, Department of Applied Physics, Aalto University, P.O. Box 13500, FI–00076 Aalto, Finland; 5 VTT Technical Research Centre of Finland Ltd, P.O. Box 1000, FI-02044 VTT, Finland; 6 Department of Physics, Lancaster University, Lancaster, LA1 4YB UK; 7 Present Address: Johns Hopkins University Applied Physics Laboratory, 11100 Johns Hopkins Road, Laurel, MD 20723 USA

**Keywords:** Ultracold gases, Bose-Einstein condensates, Quantum fluids and solids

## Abstract

Persistent topological defects and textures are particularly dramatic consequences of superfluidity. Among the most fascinating examples are the singular vortices arising from the rotational symmetry group SO(3), with surprising topological properties illustrated by Dirac’s famous belt trick. Despite considerable interest, controlled preparation and detailed study of vortex lines with complex internal structure in fully three-dimensional spinor systems remains an outstanding experimental challenge. Here, we propose and implement a reproducible and controllable method for creating and detecting a singular SO(3) line vortex from the decay of a non-singular spin texture in a ferromagnetic spin-1 Bose–Einstein condensate. Our experiment explicitly demonstrates the SO(3) character and the unique spinor properties of the defect. Although the vortex is singular, its core fills with atoms in the topologically distinct polar magnetic phase. The resulting stable, coherent topological interface has analogues in systems ranging from condensed matter to cosmology and string theory.

## Abstract (web summary)

Topological defects and textures are universal phenomena across physics. The authors demonstrate that an initial non-singular spinor texture can be controllably transformed into a pair of singular vortices with cores filled by atoms that continuously connect distinct magnetic phases of matter.

## Introduction

Quantised vortices are topological defects with universal properties that span seemingly disparate areas of science, such as high-energy physics, superconductivity, liquid crystals, and superfluids^1^. Superfluids with internal degrees of freedom such as liquid ^3^He (ref. ^2^) and dilute-gas spinor Bose–Einstein condensates^3,4^ (BECs) may exist in diverse stable phases, characterised by different broken symmetries of the full interaction Hamiltonian. Distinct states within a given phase of matter transform into one another in several ways, such as through rotations of spin and condensate phase. As a result, a rich phenomenology emerges consisting of topological defects and textures that resemble those predicted to exist in quantum field theories and cosmology^1^.

In ordinary scalar superfluids, such as superfluid liquid ^4^He and dilute BECs with frozen internal degrees of freedom, quantised vortices are characterised by the winding of the phase of the macroscopic wave function about any closed loop encircling the vortex line^5,6^. The whole spectrum of phase values converges to the singular vortex line, at which the superfluid density vanishes.

In contrast, spin-1 condensates are described by a three-component spinor, which admits both polar (P) and ferromagnetic (FM) ground-state magnetic phases. For atoms in the FM phase, the magnitude of the spin assumes its maximum value of one^3,4^, and all of the different physical states are related to each other by spatial rotations of the spinor. The distinguishable states of the system are fully represented by the orientation of a local, orthonormal vector triad defined by the orientation of the atomic spin and rotations about it, corresponding to the elements of the group SO(3) of three-dimensional (3D) spatial rotations. Mathematical analysis^7^ of this symmetry group indicates that vorticity must be carried either by coreless, non-singular spin textures, or by quantised, singular vortices.

Quantised singular SO(3) vortices with even winding numbers have the unusual property that they are topologically equivalent to the defect-free state. When the local orientation of the vector triad describing the SO(3) vortex undergoes an even number of 2*π* rotations about an axis passing through the system, the triads can be locally and continuously reoriented, smoothly returning the system to a uniform configuration. Equivalently, joining two vortices with 2*π* winding each can cancel their net vorticity, either when they circulate oppositely or—less obviously—when they wind in the same sense. This essential property has been attributed to Dirac as his eponymous belt trick, a demonstration in which two 2*π* twists of a belt in the same direction may return it to its original configuration^8^; but the concept also makes an appearance in diverse artistic contexts such as the Balinese candle dance. The significance of the belt trick to our work is that vortices with an odd number of 2*π* rotations of the vector triad are all equivalent to one another but not to the defect-free state.

In light of their peculiar properties, which have no correspondence in scalar quantum fluids, singular SO(3) vortices have attracted considerable attention in several different contexts. They have previously been described and indirectly detected in the superfluid liquid ^3^He-*A* phase^2,9,10^, where their direct visualisation is challenging. In spin-1 BECs, they have been studied theoretically as the unique class of singular vortices in the FM phase^11–14^. Of particular significance is the fact that, although the superfluid density in the FM phase must vanish along the line where the triad orientation is ill-defined, the singular vortex can lower its energy by developing a superfluid core consisting of atoms in the spinless P phase that are excited out of the FM ground-state manifold^12,14,15^. This phenomenon has been observed experimentally in the spontaneous vorticity of randomly appearing singular SO(3) defects in quasi-two-dimensional (2D) condensates during a rapid non-equilibrium phase transition^16^, where the filled vortex cores were detected indirectly by their lack of longitudinal magnetisation. More recently, atomic condensates subjected to momentum-dependent artificial gauge potentials were shown to support filled-core vortices^17^ closely related to those studied in our work. Related but topologically different half-quantum vortices have also been observed in the P phase in a quasi-2D BEC^18^. Despite these efforts, the controlled creation of singular SO(3) vortices remains an experimental challenge.

**Fig. 1:**
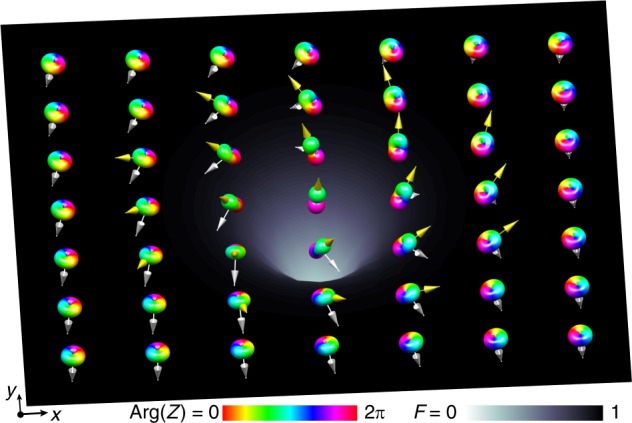
Numerical simulation of a singly quantised singular SO(3) vortex. The symmetry of the local spinor order parameter is illustrated by a graphical representation of the surface of |*Z*(*θ*, *ϕ*)|^2^, with the colour indicating Arg(*Z*), where $Z(\theta ,\phi ) = \mathop {\sum}\nolimits_{m = - 1}^{ + 1} {Y_{1,m}} (\theta ,\phi )\zeta _m$ corresponds to an expansion of the spinor in terms of spherical harmonics *Y*_1,*m*_(*θ*, *ϕ*), such that (*θ*, *ϕ*) define the local spinor orientation. Outside the vortex core the order parameter reaches the SO(3) symmetric ferromagnetic phase. Inside the core it continuously connects with the nematic order parameter of the polar phase at the vortex line singularity, forming a coherent topological interface. The interpolation of the order parameter across the interface is readily apparent in the vanishing magnitude of the spin vector $\langle {\hat{\mathbf{F}}}\rangle$ (silver arrows and background surface) at the vortex line where the polar phase is determined by the ${\hat{\mathbf{d}}}$ vector (gold arrows) and the phase of the macroscopic condensate wave function. Source data are provided as a Source Data file

Here, in a striking manifestation of the topological constraints of the SO(3) order parameter, we transform a non-singular vortex that is topologically equivalent to one with a 4*π* winding of the FM order parameter into a pair of spatially-separated singular SO(3) vortices with 2*π* winding each (Fig. 1). We thereby circumvent the smooth topological unwinding permitted by Dirac’s belt trick, dividing the equivalent of a 4*π* rotation into two 2*π* rotations that, once separated, cannot individually unwind. We find experimentally that the singular FM vortex cores are filled and expanded by atoms in the P phase. This establishes the existence of a coherent topological interface^14,19^, where the order parameter continuously interpolates between the two magnetic phases within the vortex core. Such topological interfaces are universal across many areas of physics, including superfluid liquid ^3^He at the boundary between coexisting *A* and *B* phases^20,21^, early-universe cosmology and superstring theory as domain walls^22^ and branes^23^, and solid-state physics supporting exotic superconductivity^24^. Finally, we explicitly demonstrate the SO(3) character of the vortices by enacting a change of basis, which appears experimentally as a spatial separation of phase singularities in the three spinor components. Our work directly addresses the challenges of controlled creation and simple parameter tuning of a fully 3D, singular SO(3) vortex, marking the path for a detailed study and direct imaging of the underlying topological phenomena.

## Results

### Theoretical background

The macroscopic wave function of a spin-1 BEC can be written in terms of the atomic density *n* and the three-component spinor *ζ* as $\Psi ({\mathbf{r}},t) = \sqrt {n({\mathbf{r}},t)} \zeta ({\mathbf{r}},t)$. In the FM phase, we have^3^

1 $$\zeta ^{{\mathrm{FM}}} = \frac{{e^{ - i\gamma }}}{{\sqrt 2 }}\left( {\begin{array}{*{20}{c}} {\sqrt 2 e^{ - i\alpha }\cos ^2\frac{\beta }{2}} \\ {\sin \beta } \\ {\sqrt 2 e^{i\alpha }\sin ^2\frac{\beta }{2}} \end{array}} \right),$$

which can be obtained by applying a 3D spin rotation *U*(*α*, *β*, *γ*) to the representative FM spinor (1, 0, 0)^T^. Any FM spinor is thus fully specified by the three Euler angles *α*, *β*, and *γ*, corresponding to the group of rotations in three dimensions, SO(3). As a consequence, any FM state can be represented by the orientation of a vector triad defined by the condensate spin vector $\langle {\hat{\mathbf{F}}}\rangle$ ($F \equiv |\langle {\hat{\mathbf{F}}}\rangle | = 1$) and an orthogonal vector ${\hat{\mathbf{d}}}$ (Methods).

**Fig. 2:**
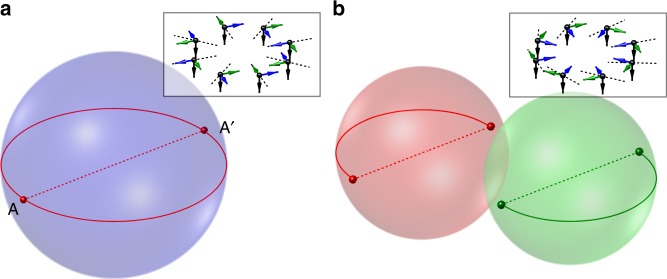
Contractible and non-contractible loops in SO(3). **a** Points inside and on the surface of the sphere represent elements of SO(3), with diametrically opposite points (e.g. *A* and *A*′) on the surface corresponding to the same element. The contractible loop on the surface of the sphere corresponds to a vortex with 4*π* winding that is continuously deformable into the vortex-free state. Such deformation amounts to enacting the Dirac belt trick. **b** The decay of the non-singular vortex into two separated singular line defects is represented by the emergence of two loops in distinct copies of the SO(3) sphere. Each loop is closed by virtue of the identification of *A* and *A*′ and cannot be contracted to a point on its own. The insets show the orientation of the ferromagnetic order parameter in real space corresponding to the points on the contractible (**a**) and non-contractible (**b**) loops, respectively. Each orthonormal triad is specified in terms of its spin direction (black arrows) and two other mutually orthonormal vectors (green and blue), with an axis of rotation given by a dashed line

The topological stability of a singular SO(3) vortex is characterised by the way closed contours encircling the defect map into the order parameter space^7^. If the order parameter space image of such a closed loop can be continuously contracted to a point, the defect is not topologically stable against transformations to the vortex-free state. The SO(3) parameter space may be represented geometrically as a solid sphere of radius *π*, where the direction of the radius vector of any point within the sphere gives an axis of rotation and its length gives the rotation angle (Fig. 2). However, *π* rotations about axes ${\hat{\mathbf{n}}}$ and $- {\hat{\mathbf{n}}}$ are equivalent, and thus diametrically opposite points on the surface must be identified. Therefore, only two topologically distinct classes of singular vortex lines exist: those that trace between identified, diametrically opposite points an even number of times, including zero; and those that trace between them an odd number of times. Mathematically, the vortex charges form the two-element group ${\Bbb Z}_2$.

Since an even number of connections between identified points always corresponds to a loop contractible to a point, the vortices in the first (even) class can be continuously deformed into the defect-free state, and those in the second (odd) class can be continuously deformed to a singly quantised, singular vortex. The essence of Dirac’s belt trick is that a 4*π* winding, with a path in parameter space that goes about the sphere once, is equivalent to the defect-free state.

### SO(3) vortex creation

**Fig. 3:**
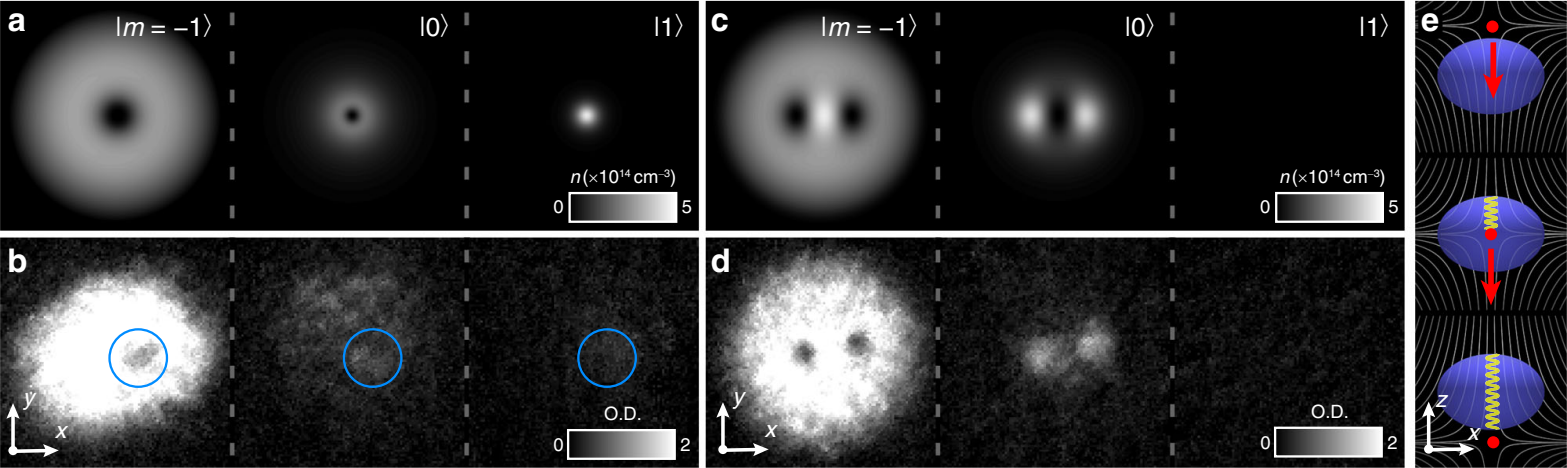
Controlled singular SO(3) vortex creation from a non-singular vortex. **a** Spinor component densities of an analytically constructed non-singular vortex state immediately after imprinting (see also Supplementary Note 1) in a cross-section through the condensate. **b** Corresponding experimental absorption images with *T*_evolve_ ≈ 0 ms for d*B*_b_/d*t* = −5 G s^−1^. The density minimum of the *m* = −1 component marks the non-singular vortex centre, and the other two components are non-zero in this region (blue circles). The creation process subjects the three spinor components to sustained differential forces, distorting the condensate and inducing non-zero densities in the *m* = 0 and *m* = 1 components distant from the vortex centre. The densities of the experimental images are expressed in terms of dimensionless optical depth (O.D.), and the field of view of each image is 219 μm × 219 μm. **c** Locally stable state after numerically simulated energy relaxation of the non-singular vortex of **b**, shown as component densities in a cross-section through the cloud. As a consequence of the SO(3) order parameter symmetry and conserved magnetisation, the non-singular vortex is unstable towards splitting into a pair of singly quantised, singular vortices, visible as density dips in the *m* = −1 component. Peaks in the *m* = 0 component at the positions of the vortices show the formation of vortex cores filled with atoms in the polar phase. **d** As **b**, but for *T*_evolve_ = 150 ms and corresponding to **c**. **e** Schematic of the imprinting process, showing the condensate (blue), magnetic field lines (grey), nodal line/axis of coreless vortex (yellow), and location of the magnetic field zero (red dot) at three sequential instants in time. The non-singular vortex is created by incompletely adiabatic spin rotations as the location of the field zero passes through the condensate in the direction of the red arrow. Source data are provided as a Source Data file

**Fig. 4:**
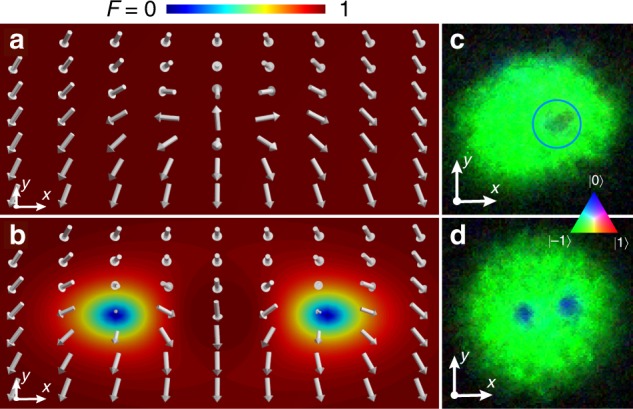
Theoretical spin textures and corresponding experimental data. **a** The characteristic fountain-like spin texture of the initial non-singular vortex, with spin magnitude one everywhere. **b** The spin texture of the relaxed vortex state. The background colour indicates spin magnitude, showing the filled vortex cores. **c**, **d** Experimentally obtained composite colour images of the corresponding structures using the data of Fig. 3, where the colours indicate the spinor components. In the absence of atoms in the *m* = 1 spinor component, pure blue represents the P phase and pure green represents the ferromagnetic phase. The field of view of **c**, **d** is 219 μm × 219 μm. Source data are provided as a Source Data file

Our primary result is a controlled creation method of a pair of singular SO(3) spinor vortices with non-trivial rotational topology from a non-singular texture. In the initial non-singular vortex—also known as a coreless vortex, baby skyrmion, or Anderson–Toulouse–Chechetkin/Mermin–Ho^2^ vortex in superfluid liquid helium—the circulation is not quantised and the spin forms a fountain-like profile that adjusts to the angular momentum of the superfluid. This characteristic fountain texture has been experimentally observed in BECs^25–27^. If the non-singular spin texture is not constrained, e.g., energetically, it can continuously deform to a vortex-free state. We find, however, that a very sharp bending of the vortex spin profile, corresponding to a strong but incomplete longitudinal magnetisation, induces an instability wherein the non-singular spin texture decays by splitting into a pair of singly quantised vortices^14^, as shown in Fig. 3a–d (see also Supplementary Note 1). Once separated, the resulting singly quantised vortices can no longer unwind on their own, thus circumventing the Dirac belt trick along the lines of Fig. 2. The decay paths of the non-singular vortex therefore include not only its unwinding by local spin rotations or departure from the condensate at its boundary^25^, but also its splitting into a pair of singly quantised SO(3) vortices that will, in turn, also ultimately leave the condensate. Numerically, a bending with magnetisation $M \lesssim - 0.3$ that is explicitly conserved is sufficient to guarantee the splitting, as shown in Figs. 3 and 4.

The splitting process of the non-singular spin texture is fundamentally different from the previously observed decay of a multiply quantised singular vortex into multiple singly quantised vortices^28–31^, in which magnetic trapping fields froze the atomic spin degree of freedom to produce a scalar BEC. In contrast, our experiment relies upon an all-optical trap that allows the atoms to retain their spinor nature. Even so, imprinting a multiply quantised singular vortex fully spin-polarises the condensate and spinor dynamics do not occur due to conservation of the maximised longitudinal magnetisation. The critical feature of our experiment is that the decay dynamics begin with an imprinted non-singular spin texture. The incomplete magnetisation ensures active spin degrees of freedom, and a spinor description is required. The relevant algebra of the line-vortex charges in our splitting process in SO(3) thus obeys the cyclic group ${\Bbb Z}_2$ with only the elements 0 and 1. Both evenly quantised and non-singular vortices are represented by the trivial element and their splitting corresponds to the group operation 0 = 1 + 1, with no counterpart in a scalar BEC.

We use time-varying magnetic fields (Fig. 3e) to initiate the creation process experimentally with a condensate initially prepared in |*m* = 1〉, where |*m*〉 denotes the *m*th spinor component. Such techniques^32,33^ have been used to prepare, e.g., non-singular^25,27,34^ and multiply quantised vortices^29^, as well as monopoles^35^, skyrmions^36^, and knots^37^.

Controlled creation of singular vortices in scalar BECs^38,39^ and continuous textures in spinor systems^26^ have also been achieved using phase imprinting methods. In our experiment the atoms experience an applied magnetic field described by

2 $${\mathbf{B}} = B_{\mathrm{b}}(t){\hat{\mathbf{z}}} + b_{\mathrm{q}}\left( {x{\hat{\mathbf{x}}} + y{\hat{\mathbf{y}}} - 2z{\hat{\mathbf{z}}}} \right).$$

where *b*_q_ is the strength of the quadrupole contribution and *B*_b_(*t*) is a time-dependent bias field that shifts the location of the point at which the magnetic field vanishes (the field zero) to *z*_0_ = *B*_b_/(2*b*_q_) on the *z*-axis. We initially choose *B*_b_ such that the field zero is slightly above the condensate (see Methods) and the magnetic field is approximately uniform (Fig. 3e).

**Fig. 5:**
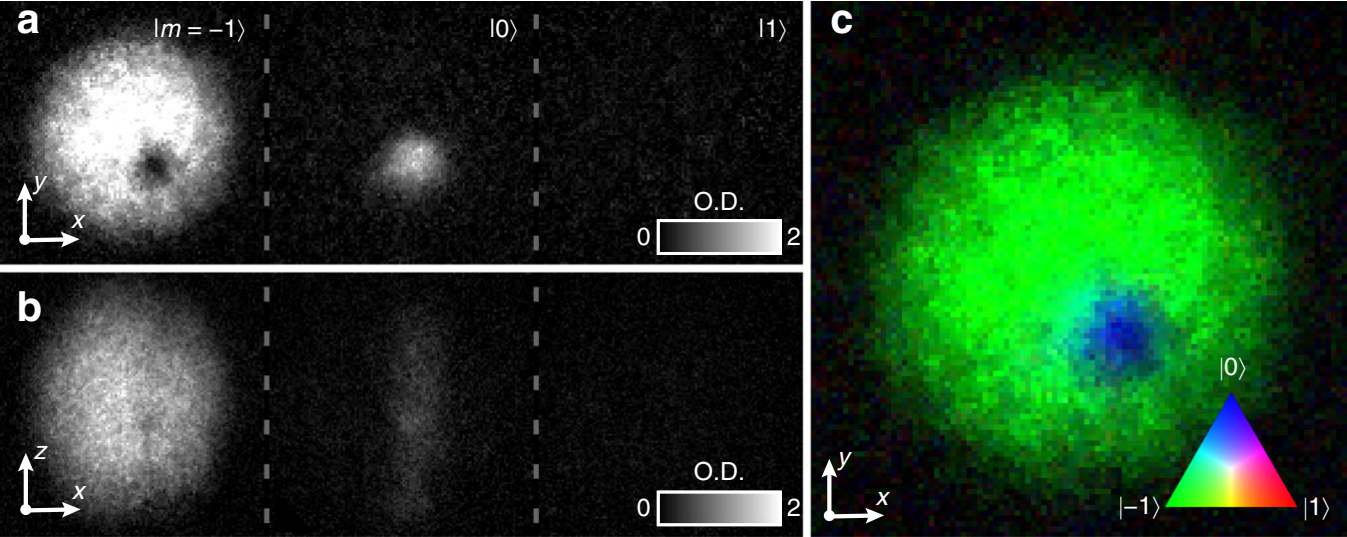
A singly quantised, singular SO(3) vortex in three dimensions. **a**, **b** Experimental absorption images of the atomic density in each spinor component from the top (**a**) and side (**b**), expressed in terms of dimensionless optical depth (O.D.), for the SO(3) vortex configuration after one of the two vortices has exited the system. The *m* = −1 component displays a vortex line, the core of which is filled with *m* = 0 atoms. The system has evolved for 1000 ms after imprinting. **c** Composite false colour image of the condensate density as viewed from the top. The field of view of each image is 219 μm × 219 μm. Source data are provided as a Source Data file

Reducing the bias field slowly induces adiabatic spin rotations as the magnetic field zero passes through the condensate from above, trailed by a 3D nodal line^35^ (Fig. 3e). At faster magnetic field ramp rates the otherwise identical experiment yields controllably incomplete adiabatic spin rotations, and results in a non-singular vortex^28,34^ with additional populations in |0〉 and |1〉 (Fig. 3a, b and Supplementary Note 1). The atoms are released from the trap after an evolution time *T*_evolve_, measured from the completion of the field ramp. Following a period of ballistic expansion they are imaged, whereupon we observe a pair of singly quantised SO(3) vortices in |−1〉 with filled cores containing atoms in |0〉, as shown in Figs. 3c, d and 4. These results agree with a numerical simulation of the locally relaxed state (Supplementary Note 1). One of these singular spinor vortices typically departs the condensate before the other, thus lowering the condensate energy^12,14^ and leaving behind a single SO(3) vortex (Fig. 5). The main dissipative sources, as in scalar BECs^6,40^, are a non-vanishing thermal cloud and potential collisions with high-temperature atoms.

### Vortex core filling and interface

**Fig. 6:**
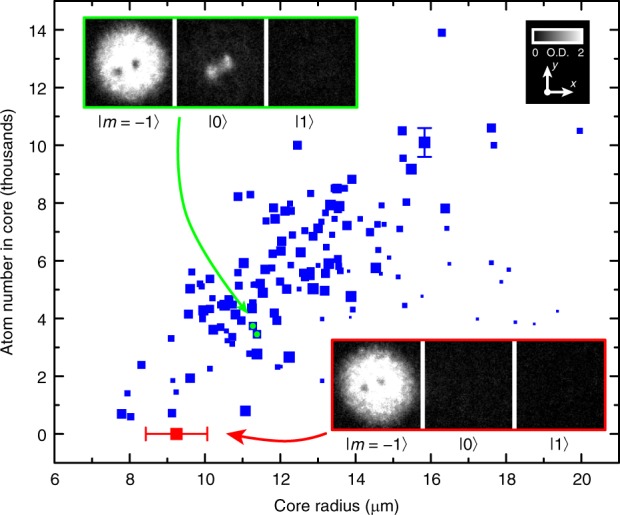
Effect of the core atoms on the size of the core. The post-expansion size of the vortex cores in the *m* = −1 component with unfilled (red) or filled (blue) cores. The core size depends in part on the number of |0〉 atoms within the core, which grows as |0〉 atoms accumulate there. Each blue point represents a single vortex measurement. As even empty vortex cores near the condensate boundary are enlarged, we indicate the radial position of a vortex by the size of the point, with smaller points corresponding to larger radii. A typical uncertainty in the atom number within the core is given as a vertical error bar for a single point. The red point and error bar illustrate the mean and standard deviation of a representative sample of unfilled vortex cores from more than 25 condensates, produced with a low-speed (|d*B*_b_/d*t*| < 1 G s^−1^) ramp of the magnetic bias field. The insets show typical experimental atomic density profiles from absorption imaging, expressed in terms of dimensionless optical depth (O.D.), of the three spinor components for the case of filled (upper left, and points marked with green circles) and empty (lower right) cores. The field of view of each image in the insets is 219 μm × 219 μm. Source data are provided as a Source Data file

For comparison, we also produce vortices with empty cores by reducing the ramp rate such that the spins rotate nearly adiabatically, leaving the system with unobservable populations in |0〉 and |1〉. The size of the filled vortex core is typically much larger than that of an empty core, as shown in Fig. 6. We have numerically verified that for our experimental parameters the superfluid vortex core expands at a rate similar to that of the whole condensate after the release from the trap. In the experiment, the size of the filled vortex core is a further manifestation of the topology of the spinor where the spinor interactions break the $|\langle {\hat{\mathbf{F}}}\rangle | = 1$ spin condition of the FM phase. In the ground state, the size of a filled vortex core is determined by a spin healing length^12,15^ arising only from the spin–spin interactions, which is much larger than the density healing length that limits the size of an empty core. Thus, as the condensate evolves, dissipation causes the filled vortex cores to inflate as |0〉 atoms accumulate there. We observe no corresponding growth of empty vortex cores, as also shown in Fig. 6.

Whereas the SO(3) order parameter of the FM phase may be represented by the orientation of an orthonormal vector triad, the P order parameter is characterised by an unoriented nematic axis ${\hat{\mathbf{d}}}$ together with the condensate phase (Supplementary Note 2). The filling of the vortex core thus results in an interface between regions where the superfluid order parameter breaks different symmetries. In our system the interface appears in the internal structure of the defect itself and is observed directly in the experiment as a smooth transition between the FM vortex state in the surrounding superfluid and the P phase at the vortex core (Fig. 5). A numerical simulation of this transition allows us to portray the condensate spinor graphically in terms of a spherical-harmonic expansion, *Z* (see Fig. 1). The deformation of *Z* illustrates the continuous topological interface that connects the SO(3) symmetric order parameter of the FM phase to the nematic order parameter of the P phase. Note that in the pure FM phase, the triad order parameter corresponds exactly to the orientation and argument of *Z*.

Analytically, the spinor describing the vortex and its superfluid core can be constructed as an interpolating filled-core vortex solution as in ref. ^14^,

3 $$\zeta = \frac{1}{2}\left( {\begin{array}{*{20}{c}} {\sqrt 2 e^{ - i\phi }\left( {{\mathrm{cos}}^2\frac{\beta }{2}D_ + - {\mathrm{sin}}^2\frac{\beta }{2}D_ - } \right)} \\ {{\mathrm{sin}}\,\beta \left( {D_ + + D_ - } \right)} \\ {\sqrt 2 e^{i\phi }\left( {{\mathrm{sin}}^2\frac{\beta }{2}D_ + - {\mathrm{cos}}^2\frac{\beta }{2}D_ - } \right)} \end{array}} \right),$$

where $D_ \pm = \sqrt {1 \pm F}$ represents the interpolation between the FM and P phases for *F* varying from 1 to 0, respectively. The azimuthal angle around the vortex line is represented by *ϕ*, and *β* is the polar angle. The spin vector is $\langle {\hat{\mathbf{F}}}\rangle = F({\rm{sin}}\beta \hat {\boldsymbol{\rho }} + {\rm{cos}}\beta {\hat{\mathbf{z}}})$, and the unit vector orthogonal to it is ${\hat{\mathbf{d}}} = - {\rm{cos}}\beta \hat {\boldsymbol{\rho }} + {\rm{sin}}\beta {\hat{\mathbf{z}}}$, where $\hat {\boldsymbol{\rho }}$ is the radial unit vector relative to the vortex line. For *F* = 1, Eq. (3) reduces to the singular FM vortex, and for *F* = 0, the spinor represents the non-circulating P phase that occupies the vortex core.

### Spinor analysis

**Fig. 7:**
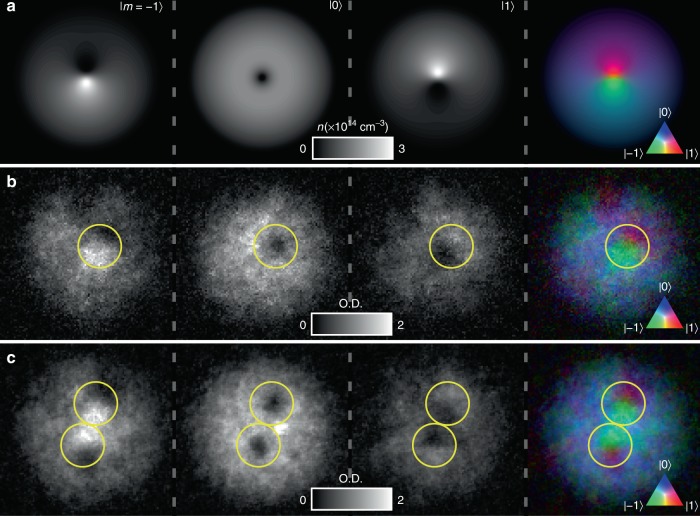
Signature of SO(3) character. **a** Spinor component densities after applying a *π*/2 spin-tip pulse to the analytically constructed singly quantised vortex, Eq. (4), corresponding to a change of spinor basis. The vortex core is at the density minimum of the |0〉 component. **b**, **c** Experimental absorption images of the atomic density, expressed in terms of dimensionless optical depth (O.D.), in each spinor component after applying a *π*/2 spin rotation for condensates containing one and two vortices, respectively. The vortex cores are identified by the density minima in the *m* = 0 component, and yellow circles are drawn around the corresponding locations in each spinor component and in the colour composite image. The false-colour composite images show alternating regions of *m* = ±1 components in the vicinity of the vortex core. The field of view of each image in **b**, **c** is 219 μm × 219 μm. Source data are provided as a Source Data file

Next, we explicitly demonstrate the SO(3) nature of the vortex. The representation of the vortex wave function as a three-component spinor depends on the choice of the spinor basis, and the order parameter symmetry dictates how the representation transforms under a change of basis. Experimentally it is more convenient to change the orientation of the spin with respect to a fixed quantisation axis by applying a radio-frequency (RF) *π*/2 pulse, which rotates the spin according to the unitary transformation *U*(0, *π*/2, *γ*_0_) where the arbitrary angle *γ*_0_ does not affect the outcome. The resulting density profiles are notably more complicated, as shown in Fig. 7. To understand these results theoretically, we assume cylindrical symmetry and neglect any small population in |1〉, leading to a qualitative model for the vortex

4 $$\zeta = \left( {\begin{array}{*{20}{c}} 0 \\ {\sqrt {1 - g(\rho )} } \\ {e^{i\phi }\sqrt {g(\rho )} } \end{array}} \right),$$

where $g(\rho ) = \rho ^2/(\rho ^2 + r_0^2)$ approximates the vortex-core profile with size parameterised by *r*_0_. The FM part of the spinor (4) in the original basis transforms as $e^{i\phi }(0,0,1)^{\mathrm{T}} \to e^{i\phi }(1/2, - 1/\sqrt 2 ,1/2)^{\mathrm{T}}$, distributing the atoms across all three components. The P part transforms as $(0,1,0)^{\mathrm{T}} \to ( - 1/\sqrt 2 ,0,1/\sqrt 2 )^{\mathrm{T}}$, splitting the atoms evenly between the |±1〉 components. Thus, after the pulse, the original atomic density distribution of the FM phase is reproduced in the |0〉 component as it only contains atoms that originated in the |−1〉 component. On the other hand, the other two components exhibit phase singularities that have shifted to different locations, leading to a split-core solution that appears to have broken the axial symmetry of the original state. This translation of the vortices after the basis transformation is a manifestation of the SO(3) symmetry of the order parameter, and indicates the presence of a line singularity about which the spin vector rotates (disgyration). After the *π*/2 rotation, one can still identify the locations of the vortices by the density minima of the atoms in the |0〉 component.

The matter wave in |±1〉 may also be interpreted as an interference between the overlapping spinor components before the spin-tip pulse. In all cases, the experimental density profiles of Fig. 7 agree well with the theoretical prediction obtained by applying a *π*/2 spin rotation to Eq. (4).

## Discussion

Our results advance the experimental and theoretical investigations of defects containing topological interfaces. Similar techniques can be used to generate half-quantum vortices, as well as vortices with coherent interfaces involving the many diverse magnetic phases observed in spin-2 spinor condensates^41–44^. The filled vortex cores themselves may be used as tracers to examine the longitudinal dynamics of the vortex lines^45^, which are otherwise difficult to discern. A further exciting extension would be to study the corresponding system in rotation where the nucleation and stability of vortices should dramatically depend on the precise value of the conserved magnetisation^14^—determining whether non-singular or singular vortices will prevail.

## Methods

### Experiment

The experimental techniques resemble those described in ref. ^35^, beginning with an optically trapped ^87^Rb condensate prepared in the FM phase (1, 0, 0)^T^ = |1〉. The optical trap frequencies are *ω*_r_ ≃ 2*π* × 130 Hz and *ω*_*z*_ ≃ 2*π* × 170 Hz in the radial and axial directions, respectively, and with an initial atom number *N* of typically 2 × 10^5^. The axial Thomas–Fermi radius of the condensate is 5 μm and the corresponding radial extent is 7 μm. The bias magnetic field *B*_b_ is controlled by a single Helmholtz coil pair, and the quadrupole magnetic field strength *b*_q_ by a second coaxial anti-Helmholtz pair. Two other pairs of coils for the *x* and *y* directions null those field components such that the field zero passes through the centre of the condensate.

The magnetic field zero is initially placed approximately 35 μm above the condensate with an initial gradient strength *b*_q_ = 4.3(4) G cm^−1^ and initial bias field *B*_b_ ≈ 30 mG. The bias field is then reduced to ~−50 mG at the rate d*B*_b_/d*t*, and then to −0.38 G over the following 10 ms. The atoms are then held in the trap for a time *T*_evolve_. An optional 8 μs, 0.266 MHz RF *π*/2 spin-tip pulse is applied immediately afterwards. At the conclusion of the experiment the quadrupole field and the optical trap are extinguished. A brief exposure to a magnetic field gradient of 70 G cm^−1^ during the 23 ms expansion separates the spinor components horizontally, after which they are imaged absorptively along the *y*- and *z*-axes in a 0.1 G field aligned with the *z*-axis. Atom loss during the experiment, both during the ramp and during the subsequent evolution time, reduces the total number of atoms to approximately 2 × 10^5^ at the time of imaging.

Reducing the bias field at the rate −0.25 G s^−1^ results in a doubly-quantised vortex in |−1〉 and essentially no atoms in the other spinor components. The experiments with filled cores were conducted at higher ramp rates, between −4 and −6 G s^−1^. Ramp rates exceeding −10 G s^−1^ result in larger non-singular vortices that occupy all three spinor components. These are not observed to evolve into singular SO(3) vortices.

### Numerical model

We use experimental parameters for the Gross-Pitaevskii Hamiltonian density of the spin-1 BEC

5 $${\cal{H}} = h_0 + \frac{{c_0}}{2}n^2 + \frac{{c_2}}{2}n^2|\langle {\hat{\mathbf{F}}}\rangle |^2 - pn\langle {\mathbf{B}} \cdot {\hat{\mathbf{F}}}\rangle + qn\langle ({\mathbf{B}} \cdot {\hat{\mathbf{F}}})^2\rangle ,$$

where $h_0 = \frac{{\hbar}^{2}}{2M_a}\left| {\nabla{\Psi}} \right|^2 + V({\mathbf{r}})n$ includes the harmonic trapping potential *V*(**r**). Here *ħ* is the reduced Planck constant and *M*_*a*_ is the atomic mass. The spin is defined as the expectation value $\langle {\hat{\mathbf{F}}}\rangle = \mathop {\sum}\nolimits_{\alpha \beta } \zeta _\alpha ^\dagger {\hat{\mathbf{F}}}_{\alpha \beta }\zeta _\beta$, where ${\hat{\mathbf{F}}}$ is a vector of dimensionless spin-1 Pauli matrices. The condensate spin vector corresponding to Eq. (1) is given by $\langle {\hat{\mathbf{F}}}\rangle = {\rm{cos}}\alpha {\rm{sin}}\beta {\hat{\mathbf{x}}} + {\rm{sin}}\alpha {\rm{cos}}\beta {\hat{\mathbf{y}}} + {\rm{cos}}\beta {\hat{\mathbf{z}}}$. The FM order parameter can be defined by the orientation of two orthogonal vectors $\langle {\hat{\mathbf{F}}}\rangle$ and ${\hat{\mathbf{d}}} = ( - {\rm{sin}}\alpha {\rm{cos}}\gamma - {\rm{cos}}\alpha {\rm{sin}}\gamma {\rm{cos}}\beta ){\hat{\mathbf{x}}} + ({\rm{cos}}\alpha {\rm{cos}}\gamma - {\rm{sin}}\alpha {\rm{sin}}\gamma {\rm{cos}}\beta ){\hat{\mathbf{y}}} + {\rm{sin}}\gamma {\rm{sin}}\beta {\hat{\mathbf{z}}}$. The last two terms of Eq. (5) describe the linear and quadratic Zeeman shift of strengths *p* and *q*, respectively. The two interaction terms of strengths *c*_0_ and *c*_2_ arise from *s*-wave scattering of the atoms.

In *s*-wave scattering the only spin-flip processes are $2\left| {m = 0} \right\rangle \rightleftharpoons \left| {m = + 1} \right\rangle + \left| {m = - 1} \right\rangle$. The longitudinal magnetisation

6 $$M = \frac{1}{N}{\int} {d^3} r{\kern 1pt} n({\mathbf{r}})F_z({\mathbf{r}}),$$

where *F*_*z*_ is the *z* component of the condensate spin, is therefore approximately conserved on time scales for which *s*-wave scattering dominates. This condition is broken when the Gross–Pitaevskii equations are made dissipative, e.g., by imaginary-time evolution. We employ an algorithm to strictly restore the conservation of magnetisation^14^ throughout energy relaxation in pure imaginary time evolution and in evolution dynamics following imprinting, in which case we set time to include a small imaginary component *t* → (1 − *iη*)*t*, where *η* ~ 10^−2^. All numerical simulations are carried out using a split-step algorithm on a minimum of 128 × 128 × 128-point grid.

## Supplementary information

Supplementary Information

Source Data

## Data Availability

All relevant data sets generated during and/or analysed during the current study are available from the corresponding author upon request. The source data underlying Figs. 1 and 3–7 are provided as a Source Data file in the Zenodo repository (10.5281/zenodo.3404017)^46^.
