# Supplementary material for: Controlled creation of a singular spinor vortex by circumventing the Dirac belt trick
Source: Nat Commun. 2019 Oct 16;10:4772. doi: 10.1038/s41467-019-12787-1 (PMC6795882; doi:10.1038/s41467-019-12787-1)
Supplement: Supplementary file 1 — Supplementary Information [file 41467_2019_12787_MOESM1_ESM.pdf]

Controlled Creation of a Singular Spinor Vortex by Circumventing the Dirac Belt Trick

Weiss et al.

Supplementary Notes

Supplementary Note 1 | Theoretical details of the $\mathrm{SO}(3)$ **vortex creation process.** A non-singular vortex with longitudinal magnetisation M can be written as

$$\zeta = \frac{1}{2}\begin{pmatrix} 1 + Mf(\rho) \\ \sqrt{2}e^{i\phi}\sqrt{1-[Mf(\rho)]^2} \\ e^{2i\phi}[1 - Mf(\rho)] \end{pmatrix}, \tag{1}$$

where ϕ is the azimuthal angle around the vortex line. The condensate spin is $\langle \hat{\mathbf{F}} \rangle = \sqrt{1-[Mf(\rho)]^2}\hat{\boldsymbol{\rho}} + Mf(\rho)\hat{\mathbf{z}}$ and the profile function $f(\rho) = \cos(\beta)/M$ satisfies $\frac{1}{N}\int d^3r\, n(\mathbf{r}) f(\rho) = 1$. The analytically constructed spinor wave function for the non-singular vortex used in Figs. 3a and 4a was obtained from supplementary equation (1) and embedded into the ground-state density profile of the condensate.

In these expressions $\beta(\rho)$ is the second Euler angle appearing in equation (1), which in this case increases monotonically from zero on the vortex line to π at the boundary to define a fountain-like spin texture. For strongly negative M, corresponding to $\beta(\rho)$ increasing rapidly, a large proportion of the condensate resides in the $|-1\rangle$ component. The two extremes of magnetisation correspond to constant $f(\rho)$: For $M = 1$, $f(\rho) = 1$, to give the vortex-free state $\zeta = (1, 0, 0)^{\mathrm{T}}$. For $M = -1$, on the other hand, we obtain the doubly-quantized vortex $\zeta = (0, 0, e^{2i\phi})^{\mathrm{T}}$ with its associated instability towards splitting. The two limits can be continuously connected through a family of functions $f(\rho)$, allowing the vortex to unwind[1]. Note, however, that conservation of longitudinal magnetisation suppresses the redistribution of the atom populations between the spinor components during the free evolution of the condensate.

Using numerical energy relaxation (Methods), we find that if the vortex is prepared such that the condensate spin vector bends rapidly towards $-\hat{\mathbf{z}}$ with increasing radial distance from the vortex centre, the spin interactions can no longer maintain a non-singular profile while still preserving the spinor nature of the condensate. The threshold approximately corresponds to a strong longitudinal magnetisation $M \lesssim -0.3$ that is explicitly conserved, and the vortex decays by splitting into a pair of singly quantized vortices as shown in Figs. 3 and 4. We show the state reached shortly after splitting of the initial vortex, exhibiting singular vortices with fully formed, filled cores. This state is locally stable: The singular

vortices persist for a significant period of imaginary-time evolution, but do eventually leave the condensate over a significantly longer period of relaxation. The local stability is also reflected in the long lifetime of the vortices created in our experiment.

The observed splitting of the coreless vortex represents a particular case of the instability also found in supplementary Ref. 2. In imprinting of a non-singular vortex, its magnetisation is a controllable parameter determined by the rate of change of the imprinting magnetic field. Our idealised model thus indicates that singular SO(3) vortices can be created by imprinting a non-singular vortex with sufficiently strong longitudinal magnetisation and allowing it to split.

Supplementary Note 2 | Magnetic Phases. The spin-1 Bose-Einstein condensate exhibits both polar (P) and ferromagnetic (FM) phases[3]. In a ^{87}Rb condensate, $c_2 < 0$ in equation (5), and hence the FM phase, which maximises $|\langle \hat{\mathbf{F}} \rangle|$, forms the ground state in a uniform system at zero magnetic field. The P phase, with $|\langle \hat{\mathbf{F}} \rangle| = 0$, appears in the cores of the singular vortices and exhibits different symmetry properties. The P order parameter, and hence also the spinor ζ^{P} in the P phase, are specified by a nematic axis $\hat{\mathbf{d}}$ together with a condensate scalar phase τ, with the property that $\zeta^{\mathrm{P}}(\hat{\mathbf{d}}, \tau) = \zeta^{\mathrm{P}}(-\hat{\mathbf{d}}, \tau + \pi)$. The P phase thus exhibits uniaxial nematic order, characterised by the continuous symmetry under spin rotations about the local, unoriented axis defined by $\hat{\mathbf{d}}$. The order parameter space is therefore $[S^2 \times \mathrm{U}(1)]/\mathbb{Z}_2$, where the factorisation by $\mathbb{Z}_2$ arises from the aforementioned nematic symmetry, which also allows the P phase to support half-quantum vortices[4].

Supplementary References

1 Ho, T.-L. Spinor Bose condensates in optical traps. *Phys. Rev. Lett.* **81**, 742–745 (1998).

2 Lovegrove, J., Borgh, M. O. & Ruostekoski, J. Stability and internal structure of vortices in spin-1 Bose–Einstein condensates with conserved magnetization. *Phys. Rev. A* **93**, 033633 (2016).

3 Kawaguchi, Y. & Ueda, M. Spinor Bose–Einstein condensates. *Phys. Rep.* **520**, 253–382 (2012).

4 Seo, S. W., Kang, S., Kwon, W. J. & Shin, Y.-i. Half-quantum vortices in an antiferromagnetic spinor Bose–Einstein condensate. *Phys. Rev. Lett.* **115**, 015301 (2015).
